# Neurologic manifestations in patients with COVID-19: A case report

**DOI:** 10.22088/cjim.11.0.557

**Published:** 2020

**Authors:** Soheil Ebrahimpour, Zeinab Mohseni Afshar, Sima Mohseni, Jila Masrour-Roudsari, Sahar Oladzade, Masomeh Bayani, Arefeh Babazadeh

**Affiliations:** 1 Infectious Diseases and Tropical Medicine Research Center, Health Research Institute, Babol University of Medical Sciences, Babol, Iran; 2 Clinical Research Development Center, Imam Reza Hospital, Kermanshah University of Medical Sciences, Kermanshah, Iran; 3 Department of Radiology, Al Jalil Hospital-Aghala , Golestan University Of Medical Sciences, Gorgan, Iran

**Keywords:** COVID-19, neurologic manifestations, case series

## Abstract

**Background:**

There are very few reports about the neurological complications of COVID-19. We describe two COVID-19 patients with neurologic presentations.

**Case Presentation:**

Herein we present neurological manifestations in 2 hospitalized patients with COVID-19. The patients showed most common symptoms of COVID-19 along with common conflicts in CT scans of lung such as ground-glass opacities (GGOs). First case revealed two episodes of generalized tonic–clonic seizures; brain CT scan in second patients revealed an extensive hypodense lesion in the left cerebellar hemisphere. All cases received supportive care, antibiotics, and antiviral medications. All cases were discharged with a good general condition.

**Conclusion:**

The current case series report the association between neurological involvements and COVID-19. Clinicians should be aware of neurologic symptoms in the setting of COVID-19, which might even be the first presentations of this infection.

In December 2019, a group of unexplained pneumonia patients has been reported in China. After a short time, new coronavirus has been named the 2019 novel coronavirus disease (COVID-19)(1). Common symptoms are fever, dry cough, dyspnea, myalgia or fatigue, and headache (2). So far, few studies demonstrate the significant incidence of neurological signs and symptoms in severe COVID-19 patients. Studies have shown neurologic manifestations of this infection categorized to central nervous system (CNS) symptoms and peripheral nervous system (PNS) symptoms (3). CNS symptoms comprised headache, dizziness, loss of consciousness, stroke, and epilepsy. Also, the most common symptoms of PNS included hyposmia, hypogeusia, and neuralgia (4). It is important to note, based on the latest guidance on COVID-19, the diagnosis of this infection is confirmed by reverse-transcription polymerase chain reaction (RT-PCR) test from throat swab samples and also finding of lesions by chest CT (Computed tomography) scans (5). Because of the increasing reports of COVID-19 patients with neurologic manifestations, it seems necessary for clinicians to be aware of this presence among any suspected or confirmed COVID-19 patient that presents with mental status change. Here, we describe two COVID-19 patients with neurologic presentations.

## Case presentation

**Case 1: **A 52-year-old male was admitted for community-acquired pneumonia with fever, cough and dyspnea to Shahid Yahyanejad Hospital, Babol, Iran. He had neither a recent travel history of China nor close contact with a COVID-19 patient. He was an otherwise healthy and non-smoker individual with no drug history.

**Figure1:**
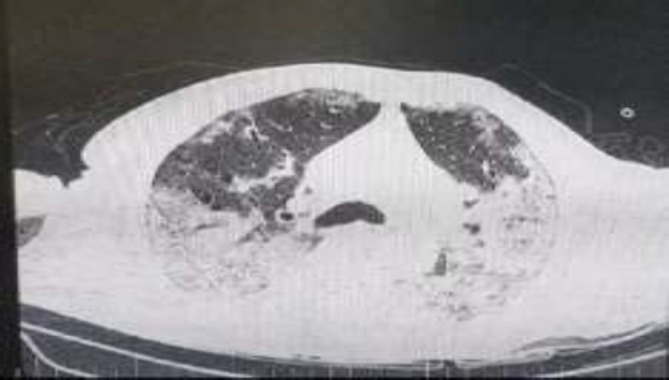
Lung CT scans of a 52-year-old male revealed bilateral multilobar peripheral ground-glass opacities (GGOs) along with interlobular septal thickening

Clinical examination revealed vital signs including temperature of 38 °C, a pulse rate of 110 beats per minute, a blood pressure of 130/80 mm Hg, a respiratory rate of 20 breaths per minute, and Oxygen saturation (SpO2) of 89% while breathing ambient air. Lung CT scan revealed bilateral multilobar peripheral ground-glass opacities (GGOs) along with interlobular septal thickening indicative of COVID-19 (figure 1). In the other words, axial non-contrast CT image showed widespread bilateral GGOs and some areas of consolidative pulmonary opacities with thickened interlobular and interlobular lines called crazy paving pattern. Also in this patient, RT-PCR assay of nasal and pharyngeal samples were positive for COVID-19. According to his laboratory data and imaging findings, he received inpatient medical and supportive treatment including supplemental oxygen for COVID-19 and his status improved thus his SpO2 reached 99% in room air, but soon after with the patient's personal consent; he was discharged. 

A few hours later, the patient returned to the emergency department with worsening dyspnea and two episodes of generalized tonic–clonic seizures. His vital signs at admission included a temperature of 38.5 °C, pulse rate of 120 beats per minute, blood pressure of 140/80 mm Hg, respiratory rate of 22 breaths per minute, and SpO2 of 98% while breathing ambient air. On neurological examination, the patient was conscious and oriented.

The cranial nerves examination was normal and his pupils were midsize and reactive to light and accommodation. His serum glucose level at the time of unconsciousness was 110 mg/dL. Other laboratory data were as follows: white blood cell (WBC), 13.00×10^9^/L with 10% lymphocytes; hemoglobin (Hb), 12.2 g/dL; platelet, 268,000/µL; C-reactive protein (CRP), 63 mg/L; interleukin-6 (IL-6), 2 pg/mL; pro-B-type natriuretic peptide (proBNP) <20 pg/mL; procalcitonin (PCT), 0.05 ng/mL; blood urea nitrogen (BUN), 11 mg/dL; creatinine (Serum), 0.6 mg/dL; sodium, 139 mEq/L; potassium, 3.8 mEq/L; magnesium, 2.2 mg/dL; phosphors, 4 mg/dL; calcium, 7.5 mg/dL; alkaline phosphatase (ALP), 170 U/L; aspartate aminotransferase (AST), 56 U/L; alanine aminotransferase (ALT), 87 U/L, and negative troponin. A brain CT scan was normal. Patients received anticonvulsant medications. A single 500 mg oral dose of chloroquine phosphate, Kaletra (lopinavir/ ritonavir) two 200 mg tablets twice daily, and azithromycin 500 mg PO daily were administered. With his condition improved and his inflammatory markers decreased, the patient was discharged.

**Case 2: **A 74-year-old male with a 3-day history of fever, anorexia, vomiting, and shortness of breath was admitted to our hospital. The patient had no history of drug and alcohol abuse and was a non-smoker. His vital signs at admission were as follows: temperature of 38.2 °C, pulse rate of 110 beats per minute, blood pressure of 130/80 mm Hg, respiratory rate of 20 breaths per minute, and SpO2 of 90% in ambient air. After a thorough workup including routine tests and computerized tomography, he received a treatment for COVID-19 and was discharged with good condition and reduced inflammatory markers. Twelve days later, he was readmitted with worsening dyspnea, palpitation, lethargy, confusion, drowsiness, blurring of vision, and generalized weakness. The initial vital signs included body temperature of 38 °C, pulse rate of 120 beats per minute, blood pressure of 140/80 mm Hg, respiratory rate of 22 breaths per minute, and SpO2 of 85% while breathing ambient air. Cranial nerves examination was normal and his pupils were midsize and reactive to light and accommodation. 

**Figure2:**
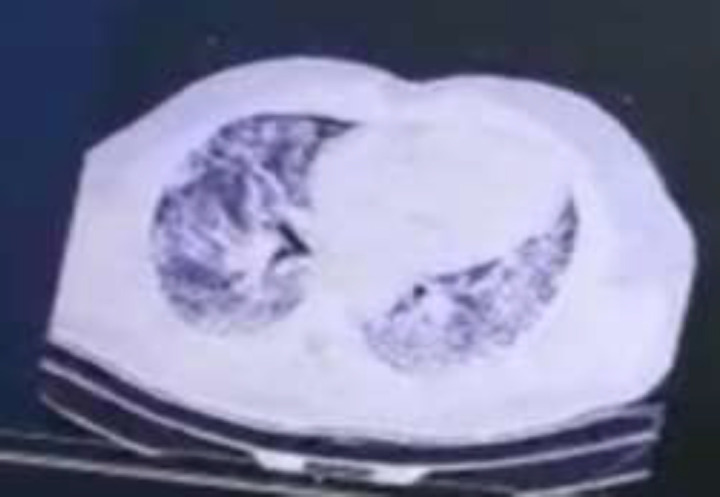
Chest CT scan of a 74-year-old male showed bilateral GGOs

**Figure 3:**
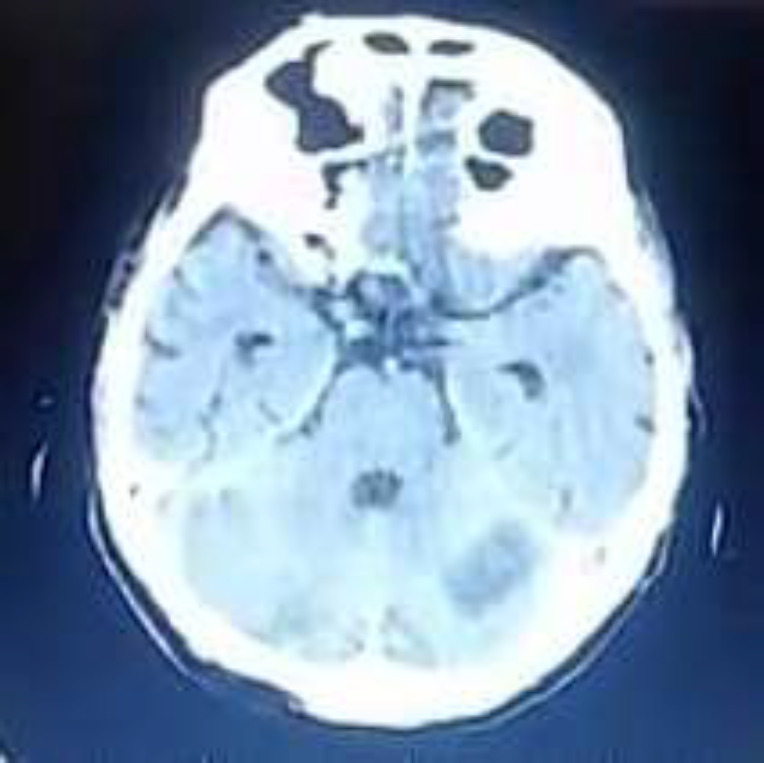
Axial unenhanced CT image of the brain showed hypodense lesion in the superior and lateral aspects of the left cerebellar hemisphere without significant mass effect

Laboratory data were as follows: blood glucose level, 102 mg/dL; BUN, 29.6 mg/dL; creatinine (Serum), 0.9 mg/dL; sodium, 138 mEq/L; potassium, 4.6mEq/L; magnesium: 1.7 mg/dL; phosphures, 4 mg/dL; calcium, 8.9mg/dL; ALP, 706 U/L; AST: 66 U/L, ALT: 53 U/L, and troponin level, 4 ng/mL. The blood sample revealed the following results: WBC count 10.4×10^9^/L with 7% lymphocytes and 91% neutrophils; CRP and erythrocyte sedimentation rate (ESR) of 128 mg/L and 57 mm/h, respectively. Chest CT scan showed bilateral GGOs which was expected due to the underlying COVID-19 infection (figure 2). The axial unenhanced CT image of the brain showed a hypodense lesion in the superior and lateral aspects of the left cerebellar hemisphere without significant mass effect (figure 3). Due to his atrial fibrillation (AF) pattern in the electrocardiogram (ECG), along with his fluctuating SpO2, the patient was admitted to the intensive care unit (ICU). Patient received heparin-based prophylaxis. A single 500 mg oral dose of chloroquine phosphate, Kaletra (lopinavir/ ritonavir) two 200 mg tablets twice daily, meropenem 1 g IV q8hr and vancomycin 1 gram q12hr were administered. After 3 days, his drowsiness diminished and he was able to tolerate oral diets. Also, his CRP level decreased to 63 mg/L. Therefore, he was transferred to the infectious diseases ward. Four days later, he developed arrhythmia, confusion and new-onset hemiplegia. At the time, WBC count was 10.00×10^9^/L with 9.5% lymphocytes; pro BNP, 8930 pg/mL; D-dimer, 7500 μg/L; CRP, 203 mg/L and PCT, 0.2 ng/mL. Anticoagulant was started for him and he was readmitted to ICU. Nine days later, his condition improved and he was discharged with an SpO2 of 95% and CRP of 30 mg/L.

## Discussion

COVID-19 infection can lead to multiple organ injuries including respiratory, gastrointestinal, and renal system. Nervous system involvement after infection with COVID-19 has been rare (6, 7). Recently, there have been reports of CNS involvement in the setting of COVID-19. As mentioned earlier, neurologic manifestations of the novel coronavirus infection may include encephalitis, toxic or metabolic encephalopathy, seizures, stroke. There are suggested different mechanisms for the etiology of neurologic injuries in the cases who suffer from viral infections such as COVID-19, including direct neuronal damage, extreme immune response through cytokine storm, unintended host immune response, and the effects of systemic illness (6). In the first case presented here, the occurrence of generalized convulsions with an imaging indicative of COVID-19 suggests the likely infection of the central nervous system by COVID-19. In any infection with new-onset convulsion, possible causes of seizure should be considered. Certain antimicrobials such as piperacillin/ tazobactam, cerebral hypoxemia, acute renal failure, and acid-base or electrolyte disturbances are some of the reasons of convulsion in the setting of severe infections (8, 9). However, none of the mentioned causes were true for our patient. In the absence of a reasonable alternative explanation for the convulsion, infection of the CNS by COVID-19 would be the most probable explanation, although supportive evidence of CFS involvement was lacking. This case shows the neuroinvasive nature of COVID-19. Fortunately, encephalitis associated with COVID-19 is usually self-limiting, as occurred in this patient. 2nd case developed hemiplegia. It has been observed that a mild course of COVID-19 may cause neurologic symptoms such as headache, mental changes, and delirium; However, serious cases have been reported to experience of disorientation, loss of consciousness, stupor, coma, and paralysis (6). The most likely mechanism of focal neurologic deficits could be ischemic brain injury induced by the virus through triggering a cytokine storm (10). 

In conclusion the reported case series affirm this important point that we should be aware of neurologic symptoms such as symptoms of the encephalitis or cerebrovascular accidents in the setting of COVID-19, which might even be the first presentations of this infection or more commonly along with respiratory symptoms.
